# MARDy: Mycology Antifungal Resistance Database

**DOI:** 10.1093/bioinformatics/bty321

**Published:** 2018-04-20

**Authors:** Anthony Nash, Thomas Sewell, Rhys A Farrer, Alireza Abdolrasouli, Jennifer M G Shelton, Matthew C Fisher, Johanna Rhodes

**Affiliations:** 1 Department of Physiology, Anatomy and Genetics, University of Oxford, Oxford, UK; 2 MRC Centre for Outbreak Analysis and Modelling, Imperial College London, London, UK; 3 Genome Sequencing and Analysis Program, Broad Institute of MIT and Harvard, Cambridge, MA, USA; 4 National Heart and Lung Institute, Imperial College London, London, UK; 5 Department of Medical Microbiology, Charing Cross Hospital, Imperial College Healthcare NHS Trust, London, UK

## Abstract

**Summary:**

The increase of antifungal drug resistance is a major global human health concern and threatens agriculture and food security; in order to tackle these concerns, it is important to understand the mechanisms that cause antifungal resistance. The curated Mycology Antifungal Resistance Database (MARDy) is a web-service of antifungal drug resistance mechanisms, including amino acid substitutions, tandem repeat sequences and genome ploidy. MARDy is implemented on a Linux, Apache, MySQL and PHP web development platform and includes a local installation of BLASTn of the database of curated genes.

**Availability and implementation:**

MARDy can be accessed at http://www.mardy.net and is free to use. The complete database can be retrieved, ordered by organism, gene and drug. Missing or new mycological antifungal resistance data can be relayed to the development team through a contribute entry form. Updates and news will be publicized via a dedicated Twitter feed: @MARDYfungi.

## 1 Introduction

The kingdom Fungi is a diverse group of eukaryotes that are essential to the sustainability of our planet. However, since the start of the Anthropocene, the occurrence of fungal disease-driven extinction of wildlife alongside human and plant infections has increased dramatically (Fisher *et al.*, 2012). Antifungal resistance has evolved directly *via* the persistent use of clinical antifungals, and indirectly *via* the application of fungicides in agriculture (Zhang *et al.*, 2017). Resistance to antifungal drugs has recently emerged as a new global challenge, raising public health concerns, a threat to food security and causes a socio-economics burden.

The Mycology Antifungal Resistance Database (MARDy) includes known resistance mechanisms of human, animal and plant fungal infections and associated drugs. There are four major classes of drugs for the treatment of fungal infections such as polyenes, azoles and echinocandins. Antifungal drug resistance is a complex phenomenon involving multiple mechanisms; MARDy includes resistance as a product of amino acid substitution, tandem repeat sequences and genome ploidy with a record to the corresponding literature. As MARDy matures we believe it will provide a valuable tool for researchers and clinicians.

The FungiDB (Stajich *et al.*, 2012) and the JGI MycoCosm (Joint Genome Initiative; Grigoriev *et al.*, 2014) are examples of dominant fungal genome databases (DBs), but these are integrated genomic and functional genomic DBs with no current capabilities to search on drug resistance mutation. Whilst CARD (The Comprehensive Antibiotic Resistance Database; Jia *et al.*, 2017) and Antibiotic Resistance Genes Database (Liu and Pop, 2009) are both comprehensive DBs of antibiotic resistance mechanisms in bacteria, and FunResDB (Weber *et al.*, 2018) is limited to mutations in the *Cyp51A* gene only, MARDy is the first DB for the kingdom Fungi and genome-wide resistance mechanisms.

The service is hosted and maintained by members of the Fungal Pathogens group in the MRC Outbreak Centre for Global Infectious Disease Analysis and Modelling. Enquires and data submission requests can be directed to the team through the link at the bottom of each page or via the Contribute page. Future releases and updates will be publicized on the website and *via* the dedicated Twitter feed @MARDYfungi.

## 2 Database content

A MySQL relational DB holds instances of resistance mechanisms and gene sequences as found in the literature. Unique entities for gene, organism, genome ploidy, amino acid substitution and tandem repeat are held in dedicated tables. Relationships between entities are constructed within linker tables: for example, an instance of an organism is linked to one or more instances of a single point amino acid substitution. For every unique gene instance (defined by a relationship between the name and locus) with a publicly available reference genome, an accompanied gene sequence is passed through the local BLASTn DB using the gene name and locus as a FASTA format identifier. A live count of genes, organisms, drugs and resistance mechanisms stored in the DB is displayed on the homepage.

## 3 Web interface

The website uses a HTML5UP template under the Creative Commons license and images were generated from our earlier work. The home screen provides a DB search panel with predictive text, a link to the complete database and a BLASTn sequence input panel. The search will successfully match a genus-species, genus or species, drug name, gene name or gene locus. The results returned in tabular form include organism name, plant/animal/human, gene name, gene locus, drug data, corresponding resistance mechanism (amino acid substitutions, tandem repeat or ploidy variation) and a link to the corresponding literature. Tables can be sorted alphabetically by clicking their table header, therefore a search by gene can readily be sorted by drug name. We plan on including multiple search capability in a future major release. A full set of search instructions have been provided in an online FAQ. A successful search is accompanied by a link to a CSV file at the bottom of the page. These are stored as files on the server and they can be saved through your browser. Saving search results is recommended, as files are left on the server for 30 days maximum. The typical time of a search query depends on Internet speed and the current load on the MARDy server. If from growing interest demand becomes too great the service will be distributed over a number of nodes in a future major release.

MARDy hosts a local distribution of BLASTn and we permit single gene sequence alignment analysis against our own DB of gene sequences. The BLASTn DB contains a wildtype genome sequence of each gene entry stored in the MARDy DB. SNP locations are highlighted along with matching gene name and organism. The complete set of BLASTn results can be downloaded in addition to the top hit gene-sequence. The gene-locus identifier from successful hits can be entered as a basic DB search query for further information. Simultaneous BLASTn queries return results without delay, however, there is scope to distribute this service over additional compute nodes if demand becomes great.

## 4 Conclusion

MARDy has the potential to become an invaluable tool for clinicians and researchers in their fight against drug-resistant fungal pathogens. We will continue to survey the literature using automated RSS feeds. In the event that growing demand sees fit, the database will be made accessible through an API.
